# CYC1 Predicts Poor Prognosis in Patients with Breast Cancer

**DOI:** 10.1155/2016/3528064

**Published:** 2016-04-28

**Authors:** Yingyan Han, Shujuan Sun, Meisong Zhao, Zeyu Zhang, Song Gong, Peipei Gao, Jia Liu, Jianfeng Zhou, Ding Ma, Qinglei Gao, Peng Wu

**Affiliations:** Cancer Biology Research Center, Tongji Hospital, Tongji Medical College, Huazhong University of Science and Technology, Wuhan 430030, China

## Abstract

Cytochrome c-1 (CYC1) is an important subunit of mitochondrial complex III. However, its role in tumor progression is unclear. We found that CYC1 was upregulated in breast tumor tissues, especially in tissues with lymph node metastasis. And higher expression of CYC1 correlates with poor prognosis in breast cancer patients using online databases and tools. Then we confirmed that CYC1 contributed to metastasis and proliferation in two highly metastatic human breast cancer cell lines. Digging into the biological function of CYC1, we found the activity of mitochondrial complex III decreased due to silencing CYC1. Then the ratio of AMP to ATP increased and AMPK was activated. Analyzing units of other mitochondrial complexes, we did not find knockdown of CYC1 expression reduced expression of any other unit of OXPHOS. We concluded that CYC1 promoted tumor metastasis via suppressing activation of AMPK and contributed to tumor growth via facilitating production of ATP. Our results indicated that CYC1 plays crucial roles in breast cancer progression and might be a predictive factor assisting future patient diagnosis.

## 1. Introduction

Breast cancer, which is one of the most commonly diagnosed cancers in women, is an epithelial malignancy of lobules or ducts [1]. The metastatic dissemination of breast cancer cells contributes to the majority of mortalities [2]. Though a large number of reports have focused on mechanisms of breast cancer metastasis, statistics show that 90% of breast cancer deaths can be attributed to metastasis [2, 3]. The ten-year survival rate of patients with diffuse metastasis is only 9% [3]. An improved understanding of metastatic dissemination of breast cancer cells was still fully wanted.

Mitochondrial biogenesis and respiration is a field helping us to know more about tumor and tumor metastasis. Warburg reported that cancer cells meet their metabolic demands through aerobic glycolysis [4, 5]. Aerobic glycolysis allows cells to use nutrient and glucose effectively and to supply abundant ATP and intermediates needed for a range of intracellular processes [6]. Also, Warburg reported that cancer cells have an irreversible injury to oxidative phosphorylation (OXPHOS) caused by increased aerobic glycolysis [4]. The irreversible injury to respiration occurs in alterations in genes expression affecting OXPHOS. However, emerging evidence has suggested that adaptive metabolic reprogramming in breast cancer cells [7, 8] and mitochondrial function played a continued vital role in the maintenance in cancer [9]. And a recent study has shown that circulating breast cancer cells exhibit a significant increase in transcript levels of mitochondrial subunits [10]. To get an improved understanding of patterns of metabolism and expression changes of mitochondrial proteins in breast tumor, we focus on the units of mitochondrial complexes.

In our previous report, CYC1 was one of the targeted genes identified by a powerful technique known as Suppression of Mortality by Antisense Rescue Technique (SMART) [11]. CYC1 (cytochrome c-1) is an important subunit of mitochondria complex III [12–14] and its mutation causes mitochondrial complex III deficiency [15]. However, the role of CYC1 in tumor progression is unclear. In this study, we found increased expression levels of CYC1 in breast cancer tissues, which was negatively correlated with clinical outcomes. In addition, expression levels of CYC1 were higher in tumor tissues with lymph node metastasis. Then we found silencing CYC1 suppressed metastasis and proliferation in two highly metastatic human breast cancer cell lines MDA-MB-231 and MDA-MB-435S cells. Silencing CYC1 expression decreased mitochondrial complex III activity and increased the ratio of AMP to ATP. Consequently, AMPK, which acts as a fuel-sensing enzyme, sensing the ratio of AMP to ATP [16], was phosphorylated and activated. Previous studies have shown that decreased activity of AMPK can promote migration and invasion in breast cancer cells [17, 18]. And decreased production of ATP contributed to suppressed proliferation of cells [5]. This study not only shows prognostic value of CYC1, but also helps us to further understand the role of CYC1 played in tumor metastasis.

## 2. Material and Methods

### 2.1. CYC1 Immunohistochemistry

Manual immunohistochemical staining was performed in order to determine CYC1 expression, using an anti-CYC1 antibody (1 : 150 dilution, Proteintech, China). A thoracic pathologist scored CYC1 staining by multiplying intensity (0–3+) and extent (0–100%) of staining via light microscopy (range 0–12).

### 2.2. Cancer Cell Lines

The MDA-MB-231 and MDA-MB-435S were obtained from the American Type Culture Collection (ATCC). MDA-MB-231 cells were cultured in L15 medium, supplemented with 10% fetal bovine serum. MDA-MB-435S cells were cultured in RPMI 1640, supplemented with 10% fetal bovine serum.

### 2.3. Gene Silencing with siRNA

CYC1 silencing experiments were performed with siRNA, sense CAGAUGUCUUAGAGUUUGAdTdT and antisense UCAAACUCUAAGACAUCUGdTdT.

### 2.4. CFSE Analysis

CFSE analysis was performed using a CFDA SE Cell Proliferation Assay and Tracking Kit (Beyotime Biotech), according to the manufacturer's instructions.

### 2.5. Cell Cycle Analysis

After treatment with CYC1 siRNA for 72 h, cells were fixed in 75% ethanol for 12 h and subsequently washed with PBS. RNase A (0.2 mg/mL) in PBS and propidium iodide were then added to the cells, in order to complete FACS cell cycle analyses.

### 2.6. Cell Migration and Invasion Assay

For wound healing assays, cells were treated with control siRNA and CYC1 siRNA. 48 h later, cells were trypsinized, and a number (4 × 10^5^ for MDA-MB-231 cells, 5 × 10^5^ for MDA-MB-435S cells) of cells from each group were plated into 6-well culture plates for 6 hours. A scratch lesion was produced using a 200 *μ*L pipette tip. Cells were then grown in complete culture medium for 48 h. We captured digital images using an inverted microscope. The transwell assays were taken using the following chamber: 8-*μ*m pore size polycarbonate (Corning). Two × 10^4^ for MDA-MB-231 cells and 3 × 10^4^ for MDA-MB-435S cells were plated into the upper compartment, coated with 100 *μ*L of Matrigel, and 600 *μ*L fresh medium was loaded into the lower compartment. After incubation for 24 h and 48 h, we removed the chambers, removed cells from the upper surface of the membranes using cotton-tipped swabs, and then stained cells using crystal violet. All experiments were performed three times in triplicate.

### 2.7. Mitochondrial Complex III Activity

The activity of complex III was assayed with the Mitochondrial Complex III Activity Detection Kit (GENMED, China), according to the manufacturer's instructions.

### 2.8. ATP and AMP Analysis

Cellular ATP content was determined using an ATP assay kit (Beyotime Biotech), according to the manufacturer's instructions. Cellular AMP content was determined using cAMP Activity Assay Kit (Biovision). These experiments were performed following treatment with control siRNA and CYC1 siRNA for 72 h.

### 2.9. RT-PCR Primers

The RT-PCR primer sets were as follows: hCYC1 5′-AGCTATCCGTGGTCTCACC-3′ and 5′-CCGCATGAACATCTCCCCATC-3′, BCS1L 5′-ACCCGTACTCAGCACCTCA-3′ and 5′-GTTCTACCCGAATCCATTTCCC-3′, UQCRC1 5′-GGGAGTGTGGATTGATGTTGG-3′ and 5′-TGTTCCCTTGAAAGCCAGATG-3′, UQCRQ 5′-CGCGAGTTTGGGAATCTGAC-3′ and 5′-TAGTGAAGACGTGCGGATAGG-3′, MTCYB 5′-GCCTGCCTGATCCTCCAAAT-3′ and 5′-AAGGTAGCGGATGATTCAGCC-3′, TTC19 5′-GCGAGCCAAGTTGAGCATTAT-3′ and 5′-GCGAGACGAAGAGCGTCAT-3′, UQCC1 5′-GGAGAAAACTGACTTCGAGGAAT-3′ and 5′-TCCAGACGTGGAGTAGGGTTA-3′, UQCC2 5′-TCAGATGTACGAGAGCTTAGCG-3′ and 5′-TGTACTCTTCCAACGACAGGC-3′, UQCR10 5′-ATCGTGGGCGTCATGTTCTTC-3′ and 5′-ATGTGGTCGTAGATAGCGTCC-3′, UQCRC2 5′-TAAGTGTGACCGCAACAAGGG-3′ and 5′-TGGTGACATTGAGCAGGAACT-3′, UQCRB 5′-GGTAAGCAGGCCGTTTCAG-3′ and 5′-AGGTCCAGTGCCCTCTTAATG-3′, UQCRFS1 5′-CGTCACCCAGTTCGTTTCCA-3′ and 5′-AGGGGTTTGCCTCTCCATTTG-3′, and UQCRH 5′-GAGGACGAGCAAAAGATGCTT-3′ and 5′-CGAGAGGAATCACGCTCATCA-3′.

### 2.10. Antibodies

The following antibodies were used: anti-CYC1 antibody (Catalog number: 10242, Proteintech, China), anti-UQCRFS1 antibody (Catalog number: 18443, Proteintech, China), anti-UQCRC2 antibody (Catalog number: 14742, Proteintech, China), anti-UQCRC1 antibody (Catalog number: 21705, Proteintech, China), anti-UQCRB antibody (Catalog number: 10756, Proteintech, China), anti-NDUFS1 antibody (Catalog number: 12444, Proteintech, China), anti-SDHA antibody (Catalog number: 14865, Proteintech, China), anti-COXIV antibody (Catalog number: 11242, Proteintech, China), anti-OSCP antibody (Catalog number: 10994, Proteintech, China), anti-AMPK antibody (Catalog number: D63G4, Cell Signaling Technology), and anti-phospho-AMPK*α* (Thr172) antibody (Catalog number: 40H9, Cell Signaling Technology).

### 2.11. Statistical Analysis

The difference between CYC1 expression in malignant breast tumors and that in benign tumors was assessed using two-tailed Student's *t*-test. All experiments in vitro were repeated three times. Statistical significance in cell invasion assays was confirmed using two-tailed Student's *t*-test. *p* < 0.05 was considered statistically significant.

## 3. Results

### 3.1. CYC1 Is Upregulated in Breast Tumor Tissues and Correlates with Poor Clinical Outcomes

**Figure 1:**
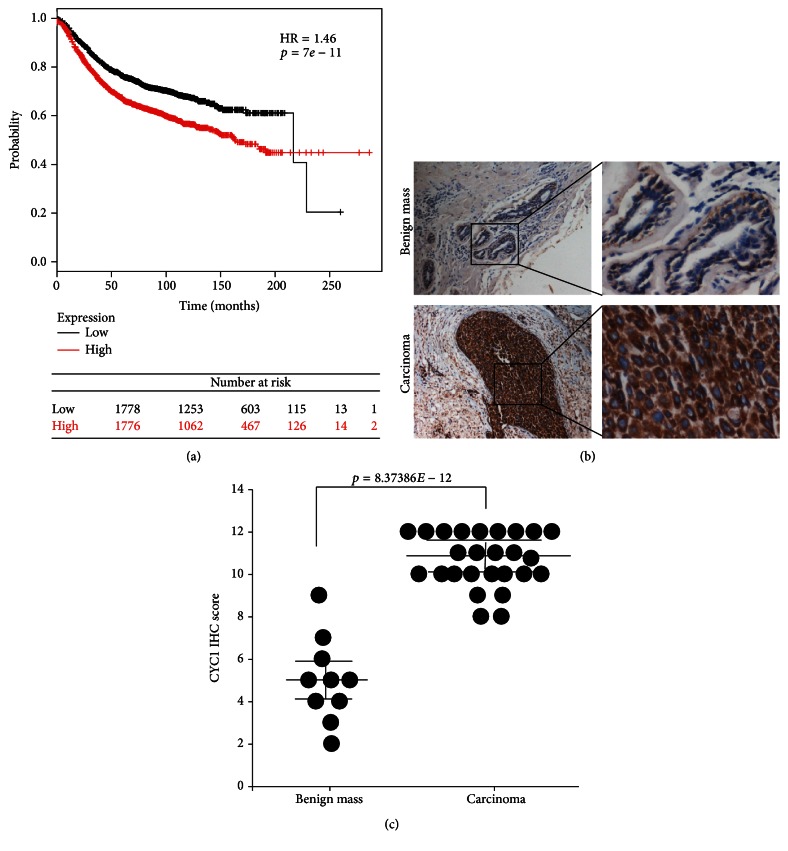
The expression of CYC1 is strongly upregulated in human malignant breast tumor tissues and is negatively correlated with patient survival. Kaplan-Meier curve was got from an integrated database and an online tool (http://www.kmplot.com/), showing the good prognostic effect of CYC1 upregulation correlated with a bad OS in breast cancer patients (*n* = 3554; use earlier release of the database: 2014 version), *p* < 0.01 (a). Low CYC1 expression in breast benign tumor (case 10, the upper row) and high CYC1 expression in malignant breast tumor (case 26, the lower row), evaluated by immunohistochemical analysis (b). IHC score of each patient was plotted as an individual dot in the chart. *p* < 0.01 (c).

In this study, an integrated database and online tool (http://www.kmplot.com/) [19] were used to determine a relationship between the level of CYC1 expression and the prognosis of breast cancer, via microarray data from 3554 breast cancer patients. The result suggested that breast cancer patients with higher expression levels of CYC1 exhibited lower survival rates (*p* < 0.01) (Figure 1(a)).

To ascertain whether CYC1 was differentially expressed in malignant breast tumors, we performed and analyzed immunohistochemistry (IHC) on human breast intraductal carcinoma, as well as benign breast tumor tissue. In this study, a total of 36 cases were involved. 26 cases were human breast intraductal carcinoma, and 10 cases were benign breast tumor. Expression of CYC1 was identified to be significantly elevated in malignant tumor tissues, relative to benign tumor tissues (Figures 1(b) and 1(c)). The clinicopathological parameters of all cases were presented in Table 1, and the result suggested that CYC1 expression levels were higher in tumor tissues with lymph node metastasis (*p* < 0.05). However, we did not find any significant correlation between CYC1 expression and clinical characteristics, such as age (*p* = 0.31), ER status (*p* = 0.885), PR status (*p* = 0.66), and HER2 status (*p* = 0.3) (Table 1).

### 3.2. CYC1 Is Responsible for Migration and Invasion in Breast Cancer Cells

**Figure 2:**
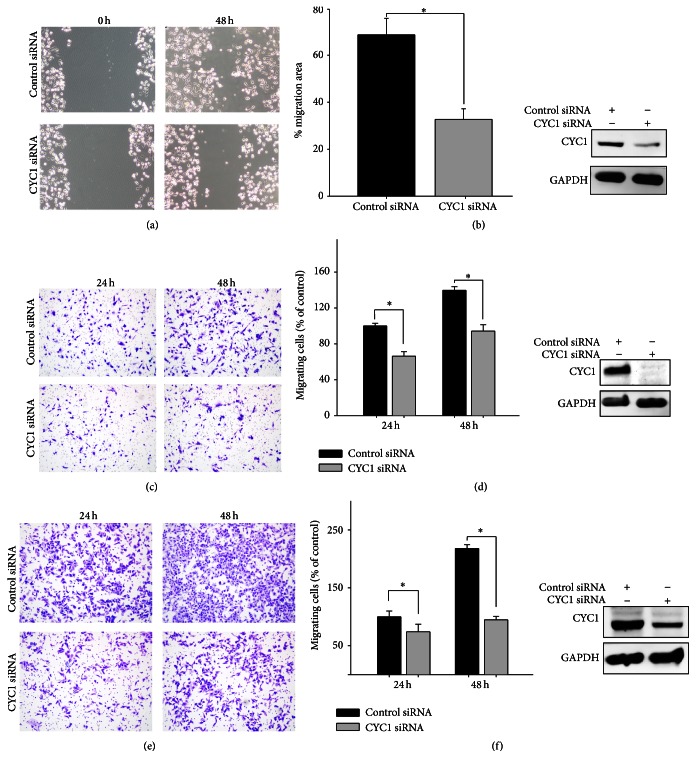
Knockdown of CYC1 expression inhibits breast cancer cells migration and invasion. The wound healing assay as monitored by optical microscopy in MDA-MB-231 and lower CYC1 expression, due to CYC1 siRNA, was confirmed by western blot (a). Quantization of migration assay showed the result of scratch assay in MDA-MB-435S, and lower expression of CYC1 in MDA-MB-435S cell was confirmed after treatment with CYC1 siRNA. (b). Transwell chamber invasion assay (c–f). Microscopic photograph of cell invasion after treatment with CYC1 siRNA in MDA-MB-231 (c) and MDA-MB-435S (e). Bar chart showed a statistical analysis of invasion assay in MDA-MB-231 (d) and MDA-MB-435S (f). ^*∗*^ *p* < 0.05.

On the basis of the results above, upregulation of CYC1 was associated with breast cancer progression and correlated to cancer cell metastasis. We firstly considered whether CYC1 expression interfered with the potential for migration and invasion. To investigate this, we performed wound healing assays and transwell assays. In wound healing assays using MDA-MB-231, the knockdown of CYC1 expression, using siRNA, dramatically reduced wound healing ability (Figure 2(a)). We received the same result when using MDA-MB-435S (Figure 2(b)). To further examine the role of CYC1 in cell invasion, we conducted a transwell invasion assay and found that silencing CYC1 expression, using siRNA, strongly decreased the invasion ability in MDA-MB-231 cells (Figures 2(c) and 2(d)) and MDA-MB-435S cells (Figures 2(e) and 2(f)). We repeated the transwell invasion assay three times after knocking down CYC1, using siRNA, and performed a statistical analysis (Figures 2(d) and 2(f)). All of these results indicated that the suppression of CYC1 inhibited breast cancer cell metastasis.

### 3.3. Silencing CYC1 Suppresses Proliferation of Breast Cancer Cells

**Figure 3:**
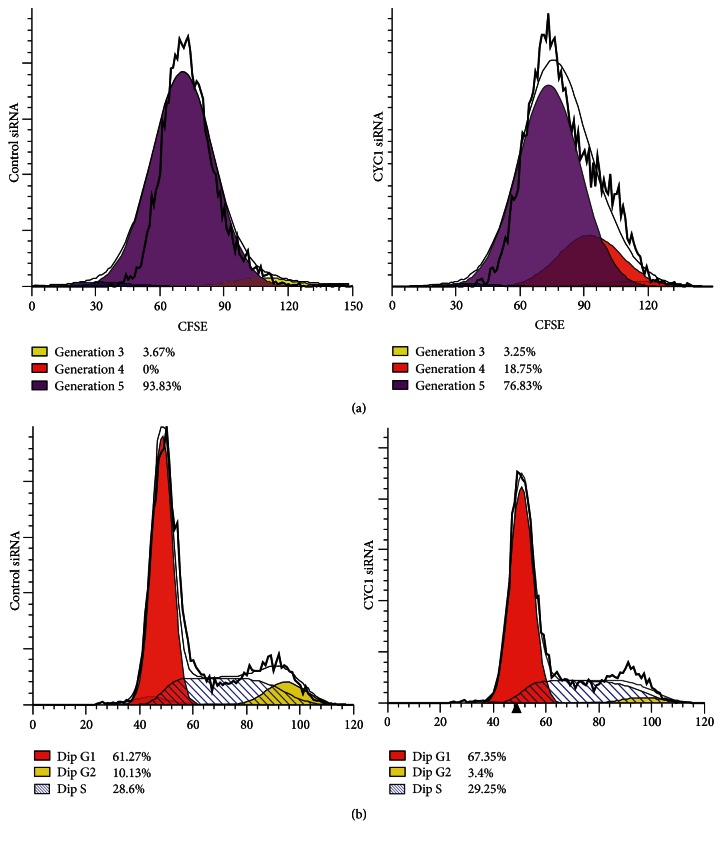
Proliferation of breast cancer cells is suppressed by CYC1 knockdown. Cell proliferation was assessed with a CFSE assay in MDA-MB-231 due to CYC1 siRNA (a). After treatment with CYC1 siRNA, cell cycle distribution of MDA-MB-231 via flow cytometry analysis was shown (b).

We investigated the influence of CYC1 expression on proliferation of MDA-MB-231, via CFSE assay. After cells were transfected with CYC1 siRNA for 4 days, 18.75% of cells were in generation 4 and only 76.83% of MDA-MB-231 cells were in generation 5. However, 93.83% of cells were in generation 5 in the control group, which means the control cells grew faster than cells treated with CYC1 siRNA (Figure 3(a)). The same result was got in MDA-MB-435S cells, but the data was not shown. Furthermore, we investigated the effect of CYC1 on cell cycle distribution via flow cytometry analysis. Compared to the control cells, knockdown of CYC1 in MDA-MB-231 cells showed more cells in G0/G1 phase and less cells in G2/M phase (Figure 3(b)). In conclusion, the proliferation of breast cancer cells was suppressed by CYC1 knockdown.

### 3.4. Knockdown of CYC1 Strongly Decreases Activity of Mitochondrial Complex III, Increases the AMP-to-ATP Ratio, and Then Activates AMPK

**Figure 4:**
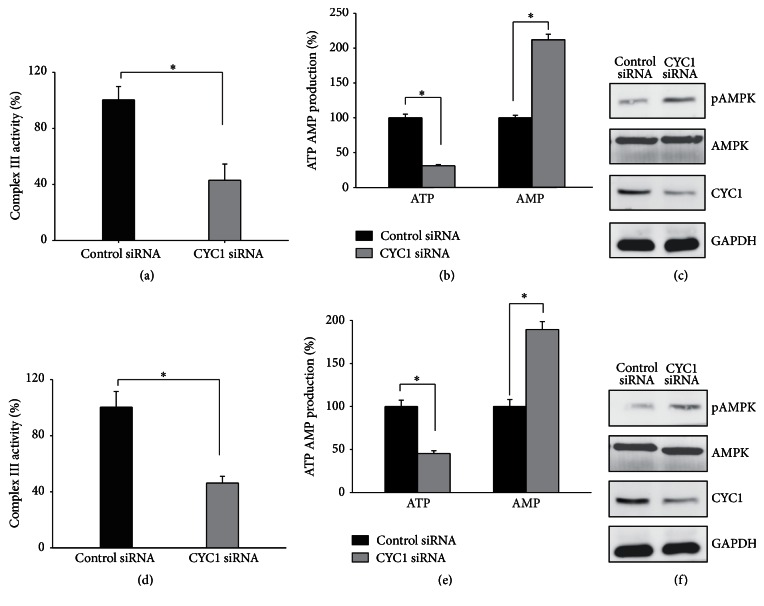
CYC1 deficiency is responsible for reduced activity of mitochondrial complex III, increased the AMP : ATP ratio, and increased phosphorylation of AMPK. MDA-MB-231 and MDA-MB-435S were treated with control siRNA and CYC1 siRNA; mitochondrial complex III activity, ATP and AMP production, and phosphorylation of AMPK were analyzed in MDA-MB-231 (a, b, c) and MDA-MB-435S (d, e, f). ^*∗*^ *p* < 0.05.

Previous studies have confirmed that CYC1 is one subunit of mitochondria complex III and is important for mitochondrial complex III activity in yeast [12, 15]. Therefore, we noted the change in complex III activity and ATP production with lower expression of CYC1. The activity of mitochondria complex III in cells treated with CYC1 siRNA was significantly reduced when compared with cells treated with control siRNA in MDA-MB-231 (Figure 4(a)) and MDA-MB-435S (Figure 4(d)). After that, we examined whether the knockdown of CYC1 affected ATP and AMP production. As observed, ATP production was reduced, while AMP production was increased, with the knockdown of CYC1 in both MDA-MB-231 (Figure 4(b)) and MDA-MB-435S (Figure 4(e)). Consistent with these findings, the SDS-PAGE analysis determined that AMPK was activated after the knockdown of CYC1 in MDA-MB-231 (Figure 4(c)) and MDA-MB-435S (Figure 4(f)).

Above all, we draw the conclusion that deficiency of CYC1 was responsible for reduced activity of mitochondrial complex III, increased ratio of AMP to ATP, and then increased phosphorylation of AMPK, which inhibit cancer cells' abilities for invasion and migration.

### 3.5. CYC1 Expression's Impact on Breast Cancer Cells Is Independent of Any Other Unit of OXPHOS

**Figure 5:**
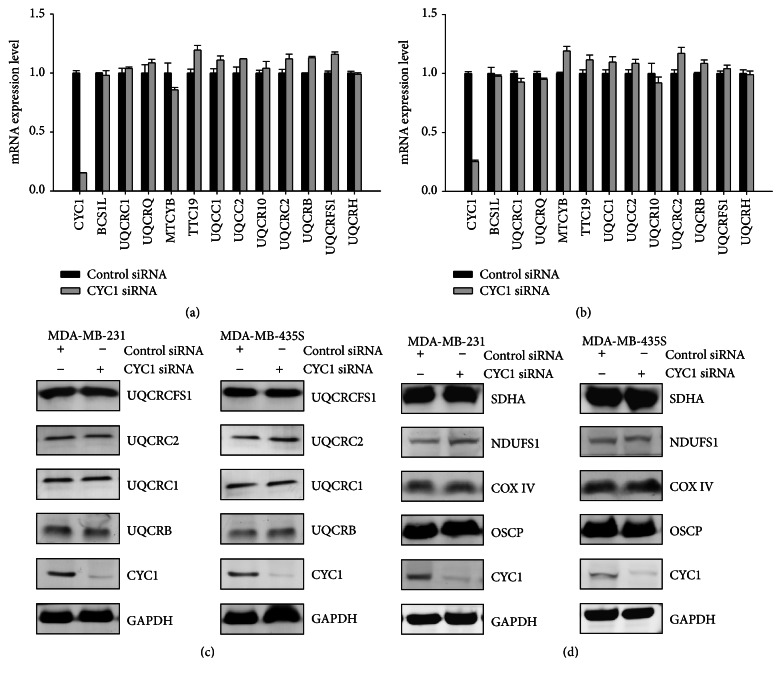
CYC1 expression is not essential for any other unit of OXPHOS. MDA-MB-231 and MDA-MB-435S cells were treated with control siRNA and CYC1 siRNA. The mRNA expression levels of the complex III subunits were identified via RT-PCR amplification in MDA-MB-231 cell (a) and MDA-MB-435S cell (b). Protein expression levels of mitochondrial complex III subunits were assessed via western blot (c). NDUFS1, SDHA, COX IV, and OSCP were, respectively, recognized as makers of mitochondrial complexes I, II, IV, and V. Protein expression levels of mitochondrial complexes I, II, IV, and V were measured by western blot (d).

Since there was reduced mitochondrial complex III activity, we further examined the impact of CYC1 expression on other units of OXPHOS, due to silencing CYC1. Firstly, the mRNA expression levels of mitochondria complex III subunits were analyzed by real-time PCR, after the knockdown of CYC1 expression, via siRNA for 72 hours. Interestingly, we did not find any significant changes in the other subunits in MDA-MB-231 (Figure 5(a)) and MDA-MB-435S (Figure 5(b)), induced by CYC1 deficiency. SDS-PAGE were assessed, and the result also demonstrated that there was no obvious influence on the other complex III subunits' protein levels in cells treated with CYC1 siRNA and control siRNA (Figure 5(c)). In conclusion, these analyses were evidence that CYC1's expression impacts mitochondrial complex III activity and ATP production but did not impact the other subunits of the OXPHOS pathway. Furthermore, protein expression levels of mitochondrial complexes I, II, IV, and V subunits also had no significant difference after treatment with CYC1 siRNA in two breast cancer cell lines (Figure 5(d)). We concluded that the consequence of CYC1 knockdown was independent of any other unit of OXPHOS.

## 4. Discussion

Rapidly proliferating tumor cells use aerobic glycolysis and this phenomenon is called the “Warburg effect” [4]. And consuming glucose rapidly may impair oxidative metabolism [4]. This standpoint is classical. However, several reports have shown mitochondrial translation and biogenesis are amplified in breast carcinoma [7, 8] and circulating breast cancer cells exhibit a significant increase in transcript levels of mitochondrial subunits [10]. In this study, we focus on a subunit of mitochondrial complex III, named CYC1. We are the first to find that CYC1 is upregulated in breast intraductal carcinoma, supporting the standpoint which suggests adaptive metabolic reprogramming in breast cancer cells, especially in those which are likely to have metastatic dissemination. In addition, using online integrated databases, we are the first to find and report that CYC1 expression is negatively correlated with breast cancer patient survival. These data showed us an improved understanding of metastatic dissemination of breast cancer cells.

The data in this report suggests that silencing CYC1 decreases the metastasis and proliferation in breast cancer cells. Digging into the mechanism of decreased tumor metastasis caused by silencing CYC1, we find reduced activity of mitochondrial complex III, increased ratio of AMP to ATP, and increased phosphorylation of AMPK. The decreased ATP production contributes to the suppressed proliferation [20]. And the activation of AMPK has the ability to inhibit cancer cell migration and invasion [18, 21]. Finally, the role of CYC1 in cancer progression is confirmed using a CYC1 siRNA in vitro transfection system, and our results can indicate that CYC1 can serve as a biomarker suggesting high probability of tumor metastasis and poor prognostic.

Targeting metabolism or mitochondrial protein is a new approach for the treatment of carcinoma, especially metastatic carcinoma [22–24]. And most of the patients who die from breast cancer die as a result of metastasis. Inhibiting the metastatic ability of cancer cells is one of the most valid approaches for treatment and for increasing the rate of survival. We have already confirmed that CYC1 is indispensable for intact function and activity of mitochondrial complex III but not essential for any other unit of OXPHOS, which means that the consequence of CYC1 knockdown was specifically triggered by itself. As a target for cancer therapy, CYC1 can avoid redundant changes, which may lead to unpredictable results and effectively inhibit metastasis in cancer cells. However, CYC1 is indispensable for intact activity of mitochondrial complex III and suppressing CYC1 may be also toxic to normal, which makes it less desirable for clinical application. But in the future, we may find new ways to overcome the toxic to normal and molecule inhibitors targeting CYC1 will be found to be effective treatment strategies for breast cancer.

## 5. Conclusion

We are the first to find that CYC1 is upregulated in breast cancer tissues and CYC1 expression is negatively correlated with breast cancer patient survival. Our results can indicate that CYC1 can serve as a biomarker suggesting high probability of tumor metastasis and poor prognosis in patients with breast cancer.

## Figures and Tables

**Table 1:** The clinicopathological parameters of all cases.

	Clinical characteristics		*n*	%	*p*
Breast malignant tumor	Gender	Male	0	0%	—
		Female	26	100%	
	Age	≤50	12	46%	0.31
		>50	14	54%	
	Pathology	Intraductal carcinoma	26	100%	—
	Histological grade	I	3	12%	—
		II	16	62%	
		III	4	15%	
	ER	Negative	13	50%	0.885
		Positive	13	50%	
	PR	Negative	13	50%	0.66
		Positive	13	50%	
	HER2	Negative	7	27%	0.3
		Positive	19	73%	
	Lymph node	Negative	13	50%	0.02^*∗*^
		Positive	13	50%	

Breast benign tumor	Gender	Male	3	30%	—
		Female	7	70%	
	Age	<50	3	30%	—
		≥50	7	70%	
	Pathology	Hyperplasia	4	40%	—
		Intraductal papilloma	1	10%	
		Cystic disease and hyperplasia	3	30%	
		Fibroadenoma	2	20%	

^*∗*^ *p* < 0.05 and this was considered statistically significant.
